# Gradient mechanical environments modulate intra-osteonal fluid flow: a three-dimensional finite element study

**DOI:** 10.3389/fbioe.2026.1722939

**Published:** 2026-02-25

**Authors:** Yu Weilun, Feng Haoyu, Gao Xu, Huang Siting, Li Xinyao, Xie Lang, Liu Xiaoxi, Yang Xiaohang

**Affiliations:** 1 Department of Orthopedics, Shanxi Bethune Hospital, Taiyuan, Shanxi, China; 2 College of Biomedical Engineering, Jilin Medical University, Jilin, Jilin, China; 3 School of Electronics and Information Technology, Sun Yat-sen University, Guangzhou, China

**Keywords:** fluid flow, mechanical microenvironment, osteon, pore pressure, pulsatile blood pressure

## Abstract

**Objective:**

Interstitial fluid flow within the osteonal lacunar-canalicular system (LCS) is crucial for osteocyte mechanotransduction and bone remodeling. This study aims to develop a three-dimensional finite element model of an osteon with gradient-varying boundary conditions to systematically investigate how mechanical loading, outer wall constraints, and pulsatile blood pressure modulate intra-osteonal fluid flow.

**Methods:**

This study constructs a three-dimensional finite element model to systematically analyze the dynamic responses of fluid flow behavior under gradient boundary conditions. Gradient parametric analyses were performed by varying: (1) axial strain amplitudes (250–5000 με) to simulate different activity levels; (2) radial displacement constraints at the outer wall (0– 0.042 μm) to represent confinement by surrounding tissues; and (3) pulsatile blood pressure amplitudes (A = 0–2.5) at the inner wall to mimic physiological to hypertensive conditions. The resulting pore pressure, fluid velocity, and fluid shear stress (FSS) distributions were analyzed.

**Results:**

All parameters exhibited axisymmetric distributions. Peak pore pressure, fluid velocity, and FSS increased nearly linearly with strain magnitude, ranging from 1.7×10^4^ to 1.4×10^5^ Pa, 1.69×10^-8^ to 3.50×10^-8^ m/s, and 0.34 to 6.5 Pa, respectively. Relaxation of outer wall constraints from fully constrained (0 μm) to fully elastic (0.042 μm) significantly reduced all three parameters. Elevated pulsatile blood pressure markedly increased intra-osteonal pore pressure (from 2.7×10^4^ to 6.5×10^4^ Pa) but had minimal effect on velocity and FSS. A subsequent multiscale validation using an explicit LCS model showed that the macro-scale poroelastic model accurately captures global trends, while local FSS within canaliculi is amplified by a factor of 1.5–2.5.

**Conclusion:**

The gradient boundary condition approach effectively quantifies the differential and synergistic effects of mechanical load, structural constraint, and vascular pressure on the osteonal fluid environment. These findings provide a quantitative framework for understanding mechanotransduction in bone and may inform clinical strategies for managing bone adaptation and disease.

## 1 Introduction

As the primary mechanical support structure of the human body, the dynamic interaction between the complex porous properties of bone and its mechanical environment profoundly influences bone tissue physiology (Zhu et al., 2021; Vanderburgh et al., 2018). The osteon, the fundamental structural unit of cortical bone, consists of a central Haversian Canal surrounded by concentric bone lamellae, forming a unique hierarchical porous network. This network is filled with interstitial fluid, which not only facilitates nutrient transport and waste removal but also serves as a critical mediator for mechanotransduction (Chang and Liu, 2022; Yu et al., 2023). Recent advancements at the interface of biomechanics and bone metabolism have highlighted the regulation of intra-osteonal fluid flow as a key pathway to understanding skeletal adaptive remodeling (Zhao et al., 2024).

An osteon typically features an outer radius of 150 μm, an inner radius of 50 μm, and a height of 1,000 μm (Yu et al., 2023), with its porous network extending from the Haversian Canal into surrounding lamellae through canaliculi (diameter 0.1–0.5 μm) that connect adjacent lacunae (Ayoubi et al., 2021). This three-dimensional interconnected structure forms a microchannel system for interstitial fluid flow, directly impacting osteocyte survival and function. Osteocytes, via cellular processes forming gap junctions with neighboring cells, establish a mechanosensitive network within the lacuno-canalicular system (Sang and Ural, 2023). Fluid flow not only supplies osteocytes with oxygen and nutrients but also transmits mechanical signals via fluid shear stress, regulating the balance between osteoblast and osteoclast activity (Wei et al., 2022).

During daily activities, human bone experiences complex mechanical loads, including axial compression, radial tension, and cyclic loading, which induce pore pressure changes within osteons through elastic deformation of the bone matrix. Moderate mechanical stimulation promotes osteoblast differentiation and matrix synthesis, while prolonged mechanical unloading (e.g., during spaceflight or bed rest) leads to bone loss (Hart, 2023; Lau et al., 2022). The core mechanism of mechanotransduction lies in osteocytes’ response to fluid flow: increased flow accelerates fluid shear stress, activating integrins and ion channels on osteocyte surfaces to trigger intracellular calcium signaling and RANKL/RANK/OPG pathways, thereby regulating bone-related gene expression (Wu et al., 2024).

The mechanical environment of osteons is composed of multiple factors, including pulsatile vascular pressure within the Haversian canal, the constraints imposed by the surrounding tissue of the bone, and axial cyclic loading (Yu et al., 2023; Smit, 2022; Kajiwara et al., 2024). Pulsatile vascular pressure arises from periodic dilation and contraction of blood vessels within the Haversian Canal, and its amplitude is modulated by systemic blood pressure status, as observed in the physiological regulation of bone blood flow (McCarthy, 2006). Surrounding tissue constraints limit the displacement of the osteonal outer wall through the elastic interaction between bone matrix and soft tissues, with experimental results showing a deformation difference of up to 0.042 μm between fully constrained and free states (Yu et al., 2023). Axial cyclic loading, generated by muscle contraction and movement, directly affects the strain distribution within osteons through its amplitude and frequency. Existing studies, however, have significant limitations in quantifying this complex mechanical environment. Traditional models often employ simplified boundary conditions of full constraint or complete freedom, neglecting the physiologically relevant gradient constraints (Yu et al., 2023; Smit, 2022; Yang et al., 2022), and the technical challenges of directly measuring internal flow characteristics due to the microstructural complexity of osteons have led to heavy reliance on numerical simulations in theoretical studies, hindering comprehensive validation of multifactorial coupling mechanisms (Yu et al., 2025).

This study aims to develop a three-dimensional finite element model of osteons with gradient-varying inner and outer boundary conditions, analyzing the effects of different vascular pressures on the inner wall and displacement constraints on the outer wall on intra-osteonal fluid flow. The “gradient boundary conditions” proposed in this work specifically refer to a series of discrete, parameterized values assigned to key mechanical environmental factors, spanning from physiological extremes to intermediate states, in order to systematically quantify their impact, rather than implying a continuous spatial gradient. This includes: (1) a gradient in loading intensity: simulating a strain range from weightlessness to destructive loading; (2) a gradient in outer wall constraint: a series of states from fully constrained by surrounding tissues to completely free; and (3) a gradient in inner wall blood pressure: pulsatile pressure variations from zero to severe hypertension. By employing this approach, this model provides a theoretical framework for systematically discussing osteonal boundary conditions under gradient variations, constructing more accurate osteonal models, and deepening the understanding of mechanical signal transduction in bone.

## 2 Materials and methods

### 2.1 Development of the osteon model and mesh generation

**FIGURE 1:**
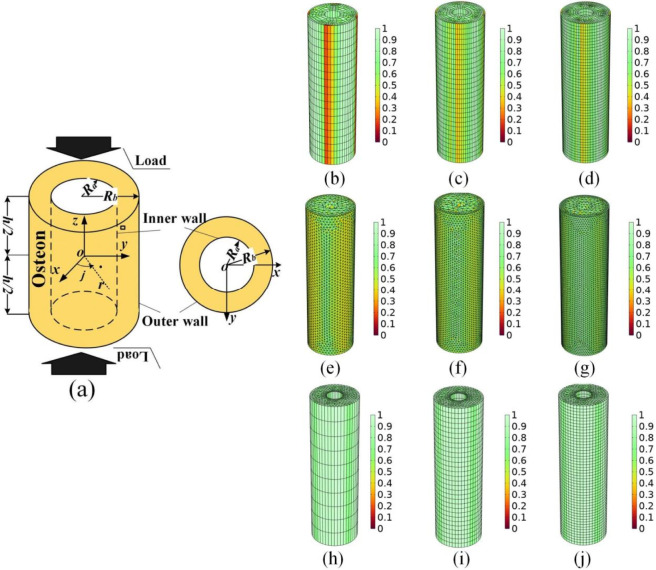
Establishment and meshing strategies for the three-dimensional osteon model. **(a)** Axisymmetric geometry of the osteon model. **(b-d)** Pure hexahedral meshes with element counts of 4,940, 10,440, and 27,900, respectively. **(e-g)** Pure tetrahedral meshes with element counts of 46,684, 72,019, and 107,879, respectively. **(h-j)** Hybrid mesh schemes with element counts of 5,780, 17,340, and 28,900.

**FIGURE 2:**
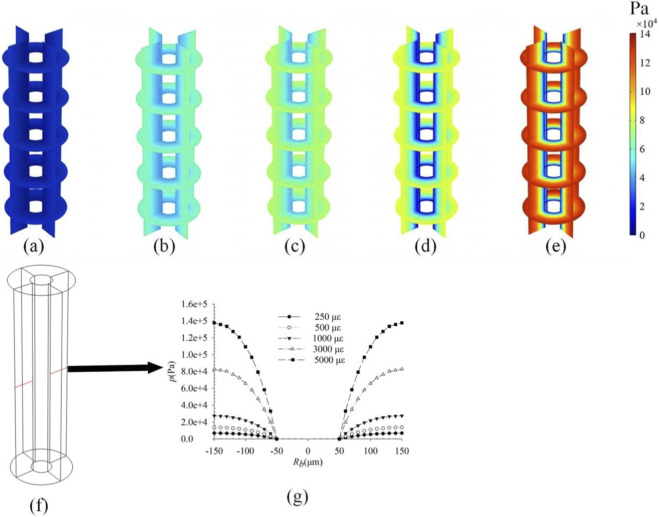
Pore pressure distributions in the osteon under strains of **(a)** 250 με; **(b)** 500 με; **(c)** 1,000 με; **(d)** 3,000 με; and **(e)** 5,000 με; **(f)** Take a line along the diameter direction of the osteon; **(g)** The variation curve of pore pressure along **(f)** under different strains.

In this study, the osteon was modeled as a three-dimensional porous medium with biomechanical characteristics. Based on the anatomical features of cortical bone microstructure, an axisymmetric geometry was established using a cylindrical coordinate system (r, θ, z) (Figure 1a). The outer radius *R*_*b*_ was set to 150 μm to simulate the typical thickness of an osteon, the inner radius *R*_*a*_ was 50 μm to represent the average diameter of the central Haversian canal, and the height h was 1,000 μm to reflect the longitudinal extension of osteons (Yu et al., 2023; Yang et al., 2022; Yu et al., 2025). These geometric parameters were derived from histological measurements of human femoral cortical bone, ensuring the model’s physiological relevance. This study aimed to investigate the effects of gradient mechanical environments (including axial loading, outer-wall constraints, and pulsatile blood pressure) on the overall distributions of pore pressure, fluid velocity, and fluid shear stress within an osteon. Therefore, we employed a macro-scale continuum poroelastic modeling approach, treating the osteon as an equivalent, homogeneous porous cylindrical medium. This simplified model was chosen based on the following considerations: (1) The study focuses on the global hydraulic response and load-transfer mechanisms of the osteon under gradient boundary conditions, rather than on the detailed local flow field within the lacunar-canalicular system (LCS). (2) Conducting systematic analysis with multiple parameters and conditions (e.g., load magnitude, levels of outer-wall constraint, blood pressure variations) within the finite element framework requires computational efficiency. The simplified model significantly reduces computational cost and enhances numerical stability. (3) Previous studies have shown that under macroscopic loading, the influence of the LCS on overall pore pressure gradients and fluid flow trends can be reasonably represented at the continuum scale through equivalent poroelastic parameters (Yu et al., 2023; Wu et al., 2020). Consequently, although the LCS network is not explicitly modeled, the present model, utilizing experimentally validated equivalent material parameters (Table 1), effectively captures the global fluid-solid coupling behavior of the osteon under complex boundary conditions and provides a reliable theoretical framework for understanding macroscopic mechanotransduction. To ensure computational accuracy and efficiency, a hybrid meshing strategy was adopted. The main body of the model (the cylindrical osteon matrix) was discretized using a structured hexahedral mesh generated by a sweeping method, while unstructured tetrahedral elements were used to fill transitional geometric regions and areas near the Haversian canal wall to accommodate complex boundary conditions and ensure mesh quality. Following generation, adaptive mesh refinement was applied, resulting in a final computational mesh comprising 28,900 elements (Figure 1j). Mesh quality was evaluated using the built-in Skewness metric in COMSOL Multiphysics. The skewness value, a standard measure in computational meshing, quantifies the deviation of an element from its ideal shape, ranging from 0 to 1. A value of 0 represents a perfectly shaped element, while 1 represents a severely degenerated one; therefore, a lower skewness value indicates better mesh quality. The software may subsequently present a derived quality score, normalized inversely, from 0 (poorest) to 1 (perfect). The assessment showed that 98% of the elements had a Skewness value greater than 0.9, validating the sound geometric quality of the mesh. To verify model stability and ensure solution independence from the mesh, a comprehensive mesh convergence study was performed. Several mesh types and density schemes were tested, including: (1) pure hexahedral meshes with element counts of 4,940 (Figure 1b), 10,440 (Figure 1c), and 27,900 (Figure 1d); (2) pure tetrahedral meshes with element counts of 46,684 (Figure 1E), 72,019 (Figure 1f), and 107,879 (Figure 1g); and (3) a hybrid mesh scheme in which triangular elements on the top surface were swept to the bottom surface, while tetrahedral elements filled the remaining volume, corresponding to element counts of 5,780 (Figure 1h), 17,340 (Figure 1i), and 28,900 (Figure 1j). By comparing the relative error in pore pressure distribution in critical regions (e.g., the Haversian canal-bone lamella junction) across different mesh densities, it was found that when using the ultimately selected hybrid scheme with approximately 17,340 elements, the rate of change in the calculated pore pressure in critical regions was less than 1%. The final mesh of 28,900 elements achieved an optimal balance between computational accuracy and resource consumption, and its convergence satisfies the accuracy requirements for engineering computations, demonstrating the numerical stability and convergence of the current meshing scheme.

**TABLE 1:** Material parameters.

*Er* (GPa) (Yu et al., 2023)	*v*_*r*_ (Yu et al., 2023)	*Ez* (GPa) (Yu et al., 2023)	*v*_*z*_ (Yu et al., 2023)	*M* (GPa) (Yang et al., 2022; Yu et al., 2025)	α (Wu et al., 2020)	φ (Yang et al., 2022; Yu et al., 2025)
15.9	0.328	20.3	0.25	38	0.12	0.05

*k* (m^2^) (Yu et al., 2023)	*μ* (Pa·s) (Yu et al., 2023)	*C*_*p*_ (1/Pa) (Yu et al., 2023; Yang et al., 2022; Yu et al., 2025)	*ρ*_*S*_ (kg/m^3^) (Yang et al., 2022; Yu et al., 2025)	*ρ*_*f*_ (kg/m^3^) (Yang et al., 2022; Yu et al., 2025)
1 × 10^−19^	1 × 10^−3^	4 × 10^−10^	2,000	1,000

### 2.2 Boundary conditions

The application of longitudinal compressive loading in this study is based on the mechanical response characteristics of bone during daily activities. As depicted in Figure 1a, the upper and lower surfaces of the osteon model are subjected to a cyclic displacement boundary condition *w*, described mathematically as follows (Yu et al., 2023; Yang et al., 2022; Yu et al., 2025).:

$$w=\pm B\left[\cos\left(2\pi ft\right)‐1\right]\left[mm\right]$$ (1)

Here, the variable *w* is the axial displacement, B denotes the displacement amplitude and f represents the loading frequency (1 Hz). This parameter configuration closely matches the loading characteristics experienced by lower limb bones during walking, inducing a 1,000 με strain in bone tissue. To simulate the dynamic compressive loading conditions experienced by osteons during activities such as weightlessness, standing, walking, jumping, and weight-bearing, we selected strain amplitudes based on physiological data: 250 με(weightlessness), 500 με(standing), 1,000 με(walking), 3,000 με(jumping), and 5,000 με(weight-bearing). The selection of these amplitudes refers to common ranges in bone mechanobiology: the peak strain during walking activity is approximately 1,000 με, which is consistent with physiological data under gait loading (Wright et al., 2024); the strain amplitudes for other activities are set based on the response thresholds of bone tissue to mechanical stimuli (e.g., strains below 200 μεmay lead to bone resorption, while strains above 3,000 με may cause pathological damage) (Wright et al., 2024), ensuring coverage of mechanical environments from low to high.

In this study, arterial pulsatile pressure (mmHg) was simulated by implementing dynamic loading boundary conditions based on the physiological characteristics of the human cardiovascular system. With reference to the blood pressure waveform of healthy adults at rest (Pfenniger et al., 2013), a piecewise function was applied to the inner wall of the osteon model, defined mathematically as:

$$Ap_{0}=A\times \left\{80+\frac{120-80}{2}\times \left\{\begin{array}{l} 0.5+0.5\cos\left(10\pi \left(t-0.1\right)\right),0<t\leq 0.1\\ 1.5-0.5\cos\left(10\pi \left(t-0.5\right)\right),0.1<t\leq 0.3\\ 0.5+0.5\cos\left(5/3\pi \left(t-0.3\right)\right),0.3<t\leq 0.9 \end{array}\right\}\right\}$$ (2)

Here, A represents the amplitude multiplier of the pulsatile pressure. The pulsatile pressure waveform was adapted from Pfenniger et al. (2013), who characterized systemic arterial pressure in a large artery. Although the absolute amplitude and frequency content may differ in Haversian microvessels due to damping and impedance changes, this waveform provides a physiologically representative template for simulating time-varying intravascular pressure. To account for uncertainties in microvascular pressure amplitude, the scaling factor A was varied from 0 to 2.5, covering a spectrum from absent pressure to severe hypertension. Given individual variations in age, physical condition, and hypertensive status, blood pressure naturally fluctuates within a physiological range. We examined the effects of blood pressure on intra-osteonal fluid flow characteristics under the following conditions: no pressure (A = 0), hypotension (A = 0.5), normal blood pressure (A = 1), mild hypertension (A = 1.5), moderate hypertension (A = 2), and severe hypertension (A = 2.5).

In this study, radial displacement constraints were defined based on the mechanical coupling characteristics between osteons and surrounding interstitial tissues. The theoretical analysis referenced here stems from our prior work (Yu et al., 2023), in which the radial displacement of a cylindrical osteon under axial load was derived from linear elasticity theory. Specifically, for a transversely isotropic material under uniaxial strain, with ν_r_ = 0.328 (as given in Table 1) and under the given axial loading condition, the theoretical radial displacement at the outer wall of a free-standing osteon was calculated to be 0.042 μm (Yu et al., 2023). Conversely, when fully constrained by surrounding tissues, the radial displacement approaches 0 μm. To simulate the gradient constraint effects in physiological states, this study adopted multistage displacement boundary conditions. This approach parametrically explores the effect of constraint degree by prescribing sinusoidal radial displacements at the outer wall, based on the theoretical range (0–0.042 µm) identified in our earlier model (Yu et al., 2023). The displacement is prescribed as:

$$u=\text{Csin}\pi t\left[um\right]$$ (3)

Here, C represents a gradient from full constraint to free deformation, taking values of 0, 0.01, 0.02, 0.03, 0.04, and 0.042. While elastic (spring) boundaries are also physiologically plausible, the prescribed displacement method allows direct control over the constraint intensity for systematic parametric analysis. Accordingly, the constraints applied to the osteon’s outer wall are defined as follows: Case 1: Full constraint (radial displacement ≈ 0 μm); Case 2: 0.01sin (πt)μm; Case 3: 0.02sin (πt)μm; Case 4: 0.03sin (πt)μm; Case 5: 0.04sin (πt)μm; Case 6: Fully unconstrained (outer wall with no displacement constraint, corresponding to free elastic deformation).

In this model, apart from the pulsatile pressure boundary condition applied at the inner wall of the osteon (*r = R*_*a*_) as described by Equation 2, all other boundaries are set as no-flow (zero normal flux) boundary conditions. This assumption is based on the physiological condition where the osteon is in close contact with adjacent structures within cortical bone, meaning there is no net fluid exchange between the osteon’s outer wall and longitudinal ends with the surrounding bone tissue or lamellae. Fluid primarily flows radially within the porous matrix between the inner and outer walls. This simplification focuses the analysis on the fluid dynamics driven by bone matrix deformation within a single osteon (Yu et al., 2023). While a simplification, this assumption is commonly adopted in studies focusing on the internal response of the osteon (Smit, 2022; Wu et al., 2020).

To investigate the influence of cement line permeability on intra-osteonal fluid flow, a concentric cylindrical shell with a thickness of 2 µm was introduced at the outer boundary (r = 150 µm) of the original cylindrical osteon model, representing the cement line (Figures 9b,c). The cement line was modeled as a porous medium thin layer possessing the same elastic modulus as the osteon body but with distinct permeability. Its permeability (k) was set as the key variable, with four logarithmically decreasing values selected for parametric study: 1 × 10^−20^ m^2^, 1 × 10^−21^ m^2^, 1 × 10^−22^ m^2^, and 1 × 10^−23^ m^2^. This gradient was designed to simulate different interfacial conditions, ranging from physiological (allowing some fluid exchange) to highly sealed (nearly impermeable). Other material parameters of the cement line (such as elastic modulus, Poisson’s ratio, porosity) were temporarily kept consistent with the osteon body (see Table 1) to isolate the effect of permeability variation.

### 2.3 Material parameters

The model employs a transversely isotropic material, with material parameters listed in Table 1 (Yu et al., 2023; Yang et al., 2022; Yu et al., 2025; Wu et al., 2020).

Here, *E*_*r*_ denotes the radial Young’s modulus, *v*_*r*_ the radial Poisson’s ratio, *Ez* the axial Young’s modulus, *v*_*z*_ the axial Poisson’s ratio, *M* the Biot modulus, α the Biot-Willis coefficient, *φ* the porosity, *k* the permeability, *u* the dynamic viscosity, *C*_*p*_ the compressibility, *ps* the solid phase density, and *pf* the liquid phase density.

### 2.4 Governing equations and model solution

Poroelasticity theory serves as the theoretical framework for investigating the mechanical behavior of porous media under external loads. This model utilizes poroelasticity theory to characterize the interaction between the solid phase (bone matrix) and fluid phase (interstitial fluid) within osteons (van Tol et al., 2020). The following governing equations describe the poroelastic behavior of bone (Yu et al., 2019):

σ=Mε−αp (4)

$$p=M\left[\xi -\text{tr}\left(\alpha \varepsilon \right)\right]$$ (5)

Here, σ denotes the total stress tensor, **ε** the total strain tensor, **M** the Biot effective stress coefficient and modulus, *p* the fluid pressure within the porous medium’s pores, and. The operator tr () represents the trace of a matrix.

Equation 6 is the momentum conservation equation of the solid skeleton, which characterizes the equilibrium between its inertial force and the divergence of the stress field:

$$\rho {\ddot{u}}^{s}-\nabla \cdot \sigma =0$$ (6)

where ρ denotes the equivalent density of the medium, ${\ddot{u}}^{s}$ represents the acceleration of the solid skeleton.

Equation 7 describes the mass conservation relationship of the liquid in porous media, given by:

$$\frac{\partial \xi }{\partial t}=-\nabla \cdot V$$ (7)

ξ the fractional change in liquid content per unit volume, ***V*** denotes the fluid velocity vector.

We use Darcy’s law to describe the quantitative relationship between the fluid seepage velocity, pressure gradient, and permeability coefficient of the medium:

$$V=-k\left(\nabla p+\rho_{f}{\ddot{u}}^{s}\right)$$ (8)

The density of the entire porous medium, *ρ*, is determined by the liquid density *ρ*_*f*_, solid density *ρ*_*s*_, and porosity *φ*, defined as: $\rho =\varphi \rho_{f}+\left(1-\varphi \right)\rho_{s}$ . Here, k=κ/μ represents the permeability coefficient tensor. Under daily physiological loads, bone is typically subjected to low-frequency cyclic loads (e.g., walking and running). Therefore, the inertial terms in the equilibrium Equation 6 and Darcy’s law (8) can be neglected. The intrinsic permeability tensor and dynamic fluid viscosity are denoted by **k** and *μ*, respectively. By substituting Equation 4 into Equation 6 and Equations 5, 8 into Equation 7, the governing Equation 9 is derived as follows:

$$\left.\begin{array}{l} \alpha \nabla p=\nabla \cdot \left(M\varepsilon \right)\\ \frac{1}{M}\frac{\partial }{\partial t}p-\nabla \cdot \left(k\nabla p\right)=-\frac{\partial }{\partial t}\left[\text{tr}\left(\alpha \varepsilon \right)\right] \end{array}\right\}$$ (9)

Using these governing equations, the pore pressure and fluid velocity of the intra-osteonal liquid can be solved by incorporating specific problem boundary conditions and material parameters.

The FSS(Fluid Shear Stress) experienced by the osteocyte and its processes was calculated using the following equation (Yu et al., 2023; Yu et al., 2025):

$$\tau =\frac{8\mu v_{i}}{d}$$ (10)

where *v*_*i*_ is the interstitial fluid velocity, d is the mean pore diameter that is calculated as follows (Yu et al., 2025):

$$d=\frac{4}{9}\sqrt{\frac{2T\sum \sum K\left(i,j\right)}{\varphi }}$$ (11)

Where T is the tortuosity of the flow path (T = 1 for straight channels), and ***K*** is the permeability tensor.

### 2.5 Parametric study design

To systematically evaluate the influence of each mechanical factor, parametric studies were conducted by varying one boundary condition while keeping the others at reference physiological values:

1. For tests at different strain levels (strains: 250, 500, 1,000, 3,000, 5,000 με), the inner wall pressure was set to normal (A = 1) and the outer wall was fully elastic.
2. For outer wall constraint tests (C = 0, 0.01, 0.02, 0.03, 0.04, 0.042 μm), the axial strain was 1,000 με and inner pressure was A = 1.
3. For pulsatile blood pressure tests (A = 0, 0.5, 1, 1.5, 2, 2.5), the axial strain was 1,000 με and the outer wall was fully elastic.

This approach ensures that the individual effect of each variable on pore pressure, fluid velocity, and shear stress can be clearly identified.

### 2.6 Micro-scale validation model

To address the limitation of the continuum poroelastic model in resolving local flow within the lacuno-canalicular system (LCS), a high-resolution micro-scale model was constructed to validate the macroscopic results (Figure 10). Based on the method described by van Tol et al. (2020) and standard osteocyte morphology, a representative volume element (RVE) containing three ideal osteocytes and their connecting canaliculi was extracted from the middle region of the osteon (R_b_ = 100 μm). The osteocyte lacunae were modeled as ellipsoids (long axis: 10 μm, short axis: 7 μm), and canaliculi were modeled as cylindrical channels (diameter: 0.6 μm) connecting the lacunae along the radial direction. The fluid flow within the LCS was governed by the Stokes equation. Boundary conditions for the micro-model, including pressure drops and flux, were directly mapped from the corresponding coordinates of the macro-scale poroelastic model under standard loading conditions (1,000 με, 1 Hz). This one-way coupling approach allows for a quantitative comparison between the volume-averaged poroelastic FSS and the explicit canalicular FSS.

## 3 Results

### 3.1 Effect of loading magnitude on fluid flow

**FIGURE 3:**
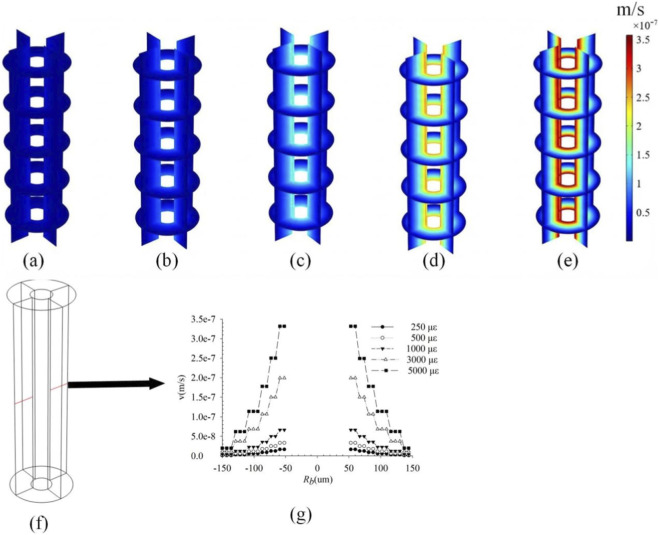
Fluid velocity distributions in the osteon under strains of **(a)** 250 με; **(b)** 500 με; **(c)** 1,000 με; **(d)** 3,000 με; and **(e)** 5,000 με; **(f)** Take a line along the diameter direction of the osteon; **(g)** The variation curve of fluid velocity along **(f)** under different strains.

**FIGURE 4:**
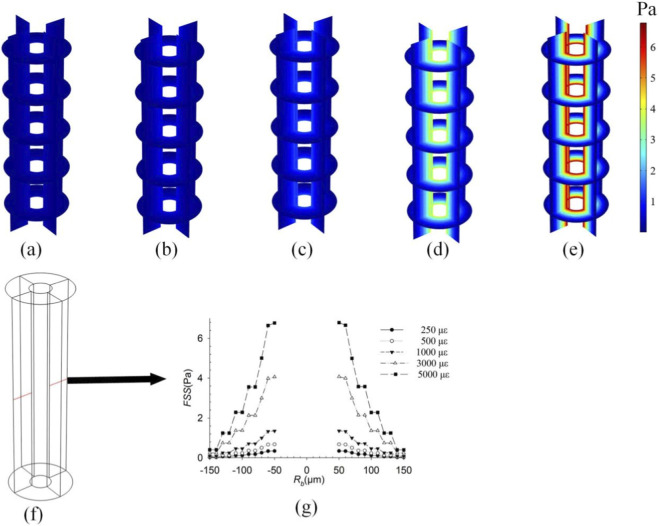
FSS distributions in the osteon under strains of **(a)** 250 με; **(b)** 500 με; **(c)** 1,000 με; **(d)** 3,000 με; and **(e)** 5,000 με; **(f)** Take a line along the diameter direction of the osteon; **(g)** The variation curve of FSS along **(f)** under different strains.

**FIGURE 5:**
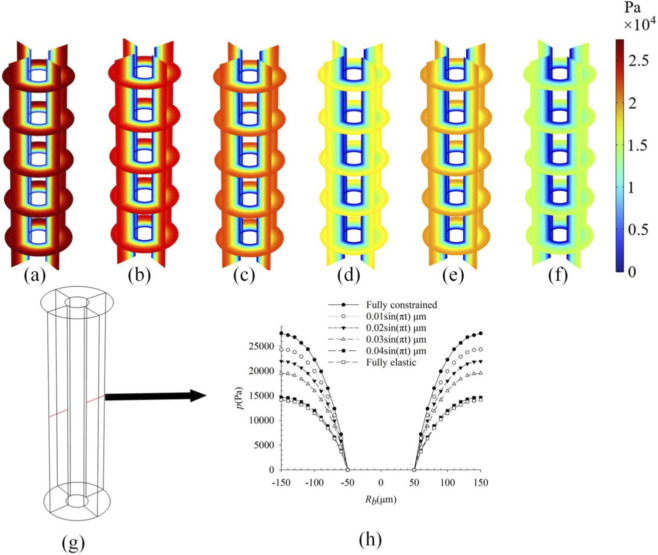
Pore pressure under different boundary conditions of the outer wall of osteons; **(a)** Fully constrained (radial displacement is approximately 0 μm); **(b)** 0.01 sin(πt) μm; **(c)** 0.02 sin(πt) μm; **(d)** 0.03 sin(πt) μm; **(e)** 0.04 sin(πt) μm; **(f)** Fully elastic (the outer wall is not constrained by displacement); **(g)** Take a line along the diameter direction of the osteon; **(h)** The variation curve of pore pressure along **(f)**.

**FIGURE 6:**
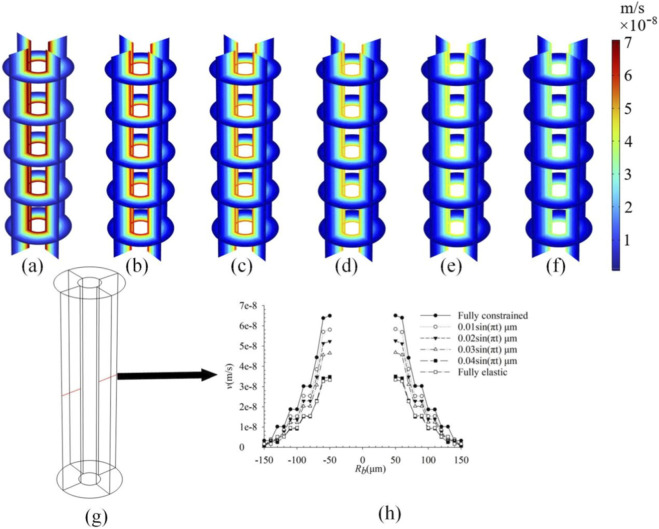
Fluid velocities under different boundary conditions of the outer wall of bone units; **(a)** Fully constrained (radial displacement is approximately 0 μm); **(b)** 0.01 sin(πt) μm; **(c)** 0.02 sin(πt) μm; **(d)** 0.03 sin (πt) μm; **(e)** 0.04 sin(πt) μm; **(f)** Fully elastic (the outer wall is not constrained by displacement); **(g)** Take a line along the diameter direction of the osteon; **(h)** The variation curve of fluid velocities along **(f)**.

**FIGURE 7:**
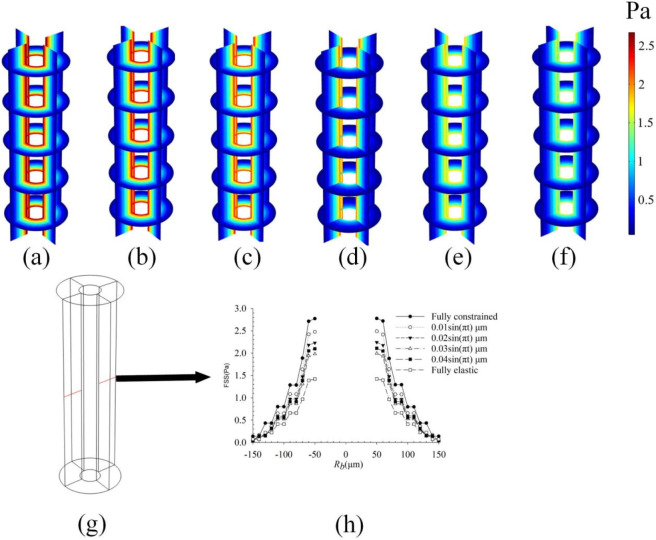
FSS under different boundary conditions of the outer wall of bone units; **(a)** Fully constrained (radial displacement is approximately 0 μm); **(b)** 0.01 sin(πt) μm; **(c)** 0.02 sin(πt) μm; **(d)** 0.03 sin(πt) μm; **(e)** 0.04 sin(πt) μm; **(f)** Fully elastic (the outer wall is not constrained by displacement); **(g)** Take a line along the diameter direction of the osteon; **(h)** The variation curve of FSS along **(f)**.

**FIGURE 8:**
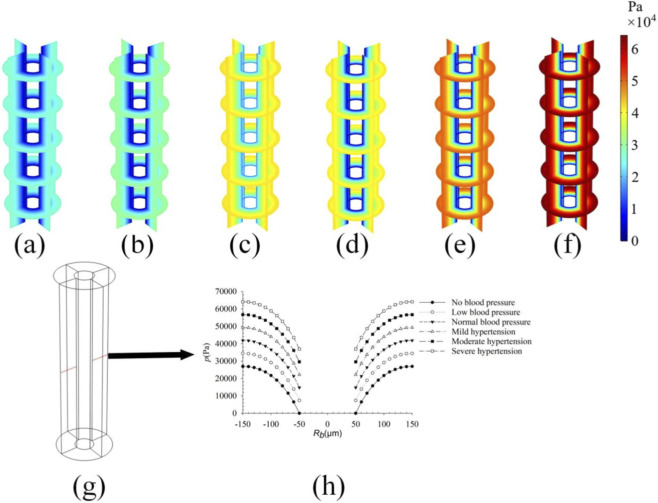
Pore pressure within bone units under different pulsatile blood pressures; **(a)** No blood pressure; **(b)** Low blood pressure; **(c)** Normal blood pressure; **(d)** Mild hypertension; **(e)** Moderate hypertension; **(f)** Severe hypertension; **(g)** Take a line along the diameter direction of the osteon; **(h)** The variation curve of pore pressure along **(f)**.

To investigate the influence of loading magnitude on intra-osteonal fluid flow, distributions of pore pressure (Figure 2), fluid velocity (Figure 3), and fluid shear stress (Figure 4) were plotted for strains of 250 με, 500 με, 1,000 με, 3,000 με, and 5,000 με induced in the osteon model. Owing to the axisymmetric geometry and loading, all parameters exhibit axisymmetric distributions. The key finding is that the peak values of all three parameters increase significantly with the applied strain. Pore pressure increased gradually with the osteon radius, reaching a minimum at the inner wall *r* = 50 μm and a maximum at the outer wall *r* = 150 μm, whereas fluid velocity and shear stress showed the opposite trend—peak values at the inner wall and decreasing toward the outer wall. These results are consistent with previous findings (Yu et al., 2023; Wu et al., 2020), validating the model’s ability to capture pressure-velocity relationships in porous bone tissue under dynamic loading. All contour plots of pore pressure, fluid velocity, and fluid shear stress presented in Figures 2–8 correspond to the time point of peak compressive strain (t = 0.25 s) within the sinusoidal loading cycle (1 Hz). This time point was selected because it represents the instant of maximum mechanical drive for fluid flow under the applied cyclic axial displacement and pulsatile pressure.

As shown in Figures 2–4, the distributions of pore pressure, fluid velocity, and fluid shear stress within the osteon under varying load magnitudes (250–5,000 µε) all exhibit axisymmetric patterns, consistent with the model’s axisymmetric geometry and loading. The simulated responses demonstrate a near-linear scaling of peak pore pressure (from 7.0 × 10^3^ Pa to 1.4 × 10^5^ Pa), peak fluid velocity (from 1.69 × 10^−8^ m/s to 3.50 × 10^−8^ m/s), and peak fluid shear stress (from 0.34 Pa to 6.5 Pa) with increasing strain—a relationship anticipated from the linear constitutive framework of Biot poroelasticity adopted in this study. This linear dose-response quantifies how efficiently macroscopic strain is converted into internal fluid-mechanical stimuli. For instance, daily activities such as walking (∼1,000 µε) and higher-intensity activities such as jumping (∼3,000 µε) can produce fluid-dynamic signals differing by an order of magnitude within the osteon. These results provide a quantitative baseline linking exercise intensity to the mechanical microenvironment experienced by osteocytes, offering a potential mechanistic explanation for the differential effects of physical activity on bone remodeling. While the linear trends are inherent to the modeling assumptions, they establish a fundamental reference for understanding how graded mechanical loads modulate intra-osteonal fluid dynamics, thereby laying a groundwork for exploring more complex, non-linear interactions under physiological and pathological conditions.

### 3.2 Effect of different boundary conditions on the osteon outer wall on fluid flow

The influence of the outer wall constraint gradient on intra-osteonal fluid flow is shown in Figures 5–7. In the model, this gradient represents a parametric sweep of the mechanical confinement provided by tissues surrounding the osteon-a confinement that *in vivo* may arise from the stiffness of the periosteum, the adjacent bone matrix, or the interstitial connective tissues. Under the linear poroelastic constitutive relations and prescribed displacement boundaries, relaxing the radial constraint from fully fixed to fully free led to a systematic, near-linear reduction in all fluid-mechanical parameters. As shown in Figure 5, pore pressure decreased progressively as the outer-wall condition changed from full constraint (Case 1: radial displacement ≈ 0 µm; peak: 2.63 × 10^4^ Pa; Figure 5a) through intermediate sinusoidal displacements of 0.01 µm (Case 2: 2.42 × 10^4^ Pa; Figure 5b), 0.02 µm (Case 3: 2.24 × 10^4^ Pa; Figure 5c), 0.03 µm (Case 4: 2.0 × 10^4^ Pa; Figure 5d), and 0.04 µm (Case 5: 1.85 × 10^4^ Pa; Figure 5e), to the fully unconstrained state (Case 6: 1.55 × 10^4^ Pa; Figure 5f). Similarly, peak fluid velocity (Figure 6) declined from 7.2 × 10^−8^ m/s under full constraint (Figure 6a) to 6.5 × 10^−8^ m/s (Figure 6b), 5.6 × 10^−8^ m/s (Figure 6c), 5.2 × 10^−8^ m/s (Figure 6d), 4.4 × 10^−8^ m/s (Figure 6e), and finally to 4.0 × 10^−8^ m/s in the free state (Figure 6f). This inverse relationship confirms that diminished radial confinement reduces the strain-derived driving force for interstitial fluid flow. Correspondingly, the fluid shear stress (FSS) distribution (Figure 7) exhibited a consistent gradient: peak FSS measured 2.58 Pa (fully constrained, Figure 7a), 2.3 Pa (Figure 7b), 2.0 Pa (Figure 7c), 1.8 Pa (Figure 7d), 1.6 Pa (Figure 7e), and 1.5 Pa (fully free, Figure 7f). This quantifies how the mechanical signal perceived by osteocytes scales directly with the degree of external mechanical support. Although the directional trend follows directly from the constitutive model, the quantitative mapping of the constraint gradient delivers novel system-level insight. It establishes, for the first time, a continuous scaling relationship between the peri-osteonal mechanical confinement which may reflect the integrated stiffness of periosteal and interstitial tissues and the amplitude of fluid-mechanical stimuli inside the osteon. This result offers a plausible micro-environmental mechanism for clinical observations: in disuse states (e.g., prolonged immobilization or weightlessness), a reduction in the effective stiffness of surrounding tissues could lower intra-osteonal FSS by approximately 42% (from the fully constrained baseline). Such an attenuation would be expected to diminish mechanotransduction signals that are essential for maintaining bone mass, thereby contributing to the progression of disuse-related bone loss.

### 3.3 Effect of different pulsatile blood pressures on the osteon inner wall on fluid flow

To investigate the influence of different pulsatile blood pressures on interstitial fluid pressure and velocity within osteons, we selected common physiological states including hypotension, normotension, mild hypertension, moderate hypertension, and severe hypertension at the osteon inner wall (Lau et al., 2022), and compared the variations in pore pressure, fluid velocity, and fluid shear stress under these conditions. Results show that pore pressure within the osteon increases with increasing pulsatile blood pressure (Figure 8), while having little to no effect on fluid velocity and shear stress. Panels (a)–(f) in Figure 8 depict pore pressure distributions under no blood pressure, hypotension, normotension, mild hypertension, moderate hypertension, and severe hypertension at the osteon inner wall, respectively. The corresponding peak pore pressures are 2.7e4 Pa, 3.5e4 Pa, 4.2e4 Pa, 5.0e4 Pa, 5.7e4 Pa and 6.5e4 Pa, indicating a direct correlation between blood pressure magnitude and intra-osteonal pore pressure across the tested physiological range.

**FIGURE 9:**
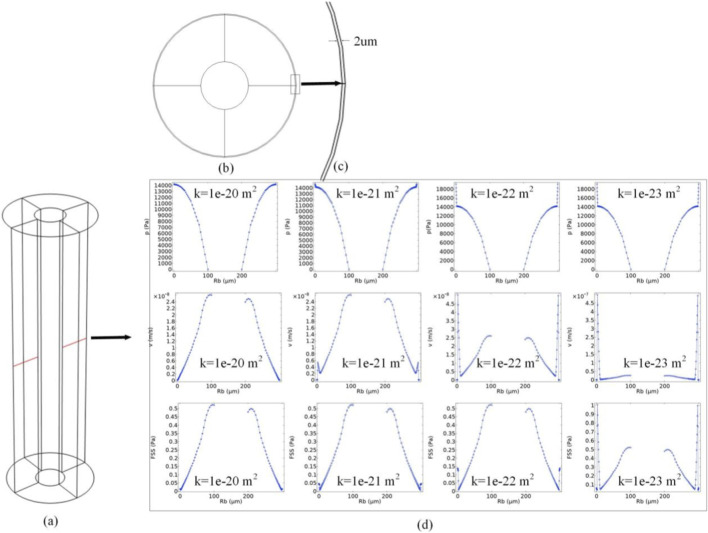
Effect of cement line permeability on osteonal fluid flow. **(a)** Schematic line profile across the osteon diameter for data extraction. **(b)** Cross-section of the osteon model with the cement line highlighted. **(c)** Magnified view of the cement line region **(b)**. **(d)** Variations in peak pore pressure, fluid velocity, and FSS for different cement line permeabilities.

Figure 9 illustrates the influence of cement line permeability on the distributions of pore pressure, fluid velocity, and fluid shear stress within the osteon. The analysis reveals that the cement line, as a critical interface between the osteon and its external environment, plays a significant regulatory role in the internal fluid mechanical environment. As the cement line permeability decreases substantially from 1 × 10^−20^ m^2^ to 1 × 10^−23^ m^2^ (Figure 9d), the intra-osteonal pore pressure increases systematically. Under low permeability conditions (e.g., k = 1 × 10^−23^ m^2^), the cement line acts almost as an impermeable flow barrier, confining the fluid generated by axial loading and pulsatile blood pressure within the osteon. This leads to higher pressure accumulation in the region near the outer wall (inside the cement line). Conversely, when the cement line has higher permeability (e.g., k = 1 × 10^−20^ m^2^), fluid can more readily cross the interface into the surrounding tissue, allowing the internal pressure gradient to dissipate and resulting in lower overall pore pressure levels. Fluid velocity and FSS exhibit trends correlated with the pore pressure changes. A highly permeable cement line allows smoother fluid outflow, leading to a reduction in the overall Darcy velocity within the osteon (Figure 9d). Correspondingly, the flow-velocity-dependent FSS also decreases. When the cement line permeability is extremely low, although the internal pressure increases, the overall fluid flow is impeded, resulting in less pronounced changes in velocity and FSS in regions far from the Haversian canal. However, in the narrow region immediately adjacent to the cement line, specific flow pattern alterations may occur due to concentrated pressure gradients.

### 3.4 Validation of osteon-scale results with explicit LCS modeling

**FIGURE 10:**
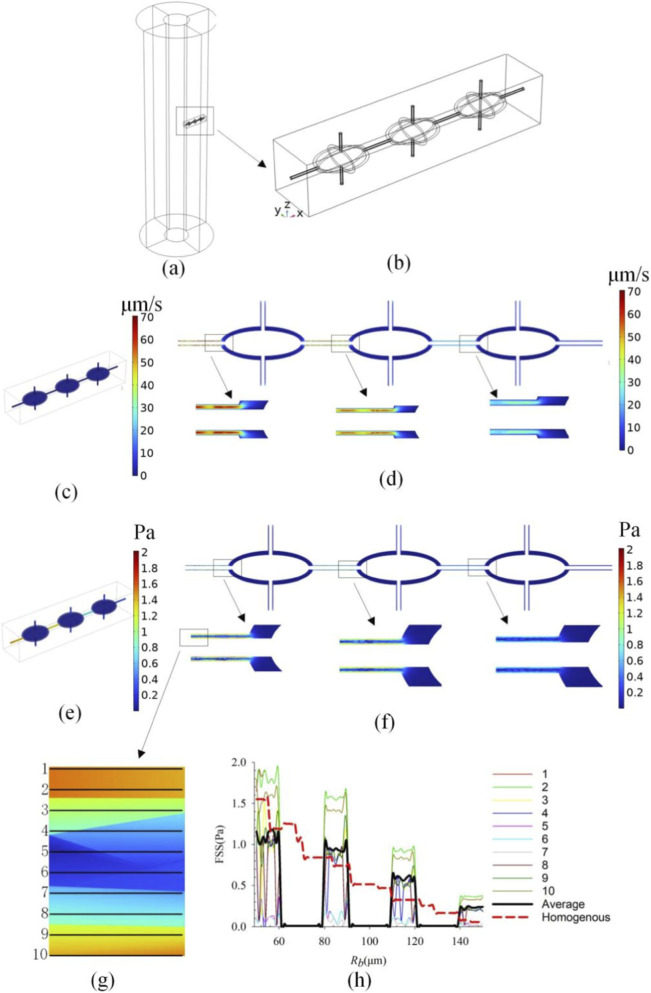
Multiscale validation of intra-osteonal fluid flow. **(a)** The location of the micro-scale representative volume element (RVE) within the macro-scale osteon model. **(b)** Geometry of the explicit LCS model, containing ideal ellipsoidal lacunae and cylindrical canaliculi. **(c)** 3D distribution of fluid velocity in the LCS. **(d)** Cross-sectional view of velocity distribution, showing flow acceleration in canaliculi. **(e)** 3D distribution of Fluid Shear Stress (FSS). **(f)** Cross-sectional view of FSS. **(g)** Schematic of the 10 data extraction paths along the canaliculi for averaging. **(h)** Quantitative comparison of FSS along the radial direction: The red dashed line represents the result from the homogeneous poroelastic model (current study), and the black solid line represents the average FSS from the explicit LCS model.

Figure 10 presents the fluid velocity and fluid shear stress (FSS) distributions within the explicit LCS micro-model and their comparison with the macro-scale poroelastic model. As shown in Figures 10c,d, the fluid velocity field in the micro-model exhibits significant heterogeneity. The velocity is markedly amplified within the narrow canaliculi (reaching peak values of 70 μm/s) while remaining low within the lacunar spaces (5 μm/s). A similar trend is observed for FSS (Figures 10e,f), where the highest shear stresses are concentrated on the canalicular walls. To quantitatively validate the poroelastic assumption, we extracted data from 10 parallel paths along the radial direction of the canaliculi (Figure 10g) and compared their average values with the results from the homogeneous poroelastic model (Figure 10h). The red dashed line represents the FSS calculated by the macro-scale poroelastic model (representing the volume-averaged FSS), while the black solid line represents the spatial average of the explicit LCS model. The comparison reveals two key findings: (1) Trend Consistency: The global gradient of the FSS predicted by the poroelastic model aligns well with the average stress drop trend in the explicit LCS model, confirming that the continuum model correctly captures the macroscopic driving forces (pressure gradients). (2) Local Amplification: The explicit LCS model reveals a “peak-and-valley” distribution. The FSS within the canaliculi is approximately 1.5–2.5 times higher than the homogeneous value predicted by the poroelastic model. This indicates that while the macro-model provides a reliable baseline, the actual mechanical stimulus perceived by osteocyte processes is amplified by the geometric constraints of the canaliculi.

## 4 Discussion and conclusion

As the fundamental structural unit of cortical bone, the osteon’s internal fluid flow plays a central role in nutrient supply to osteocytes and mechanical signal transduction. Nutrients in blood, including glucose, amino acids, and minerals, enter the Haversian system via blood vessels (Evrard et al., 2024), then diffuse through the lacuno-canalicular system with the aid of fluid flow, ultimately reaching osteocytes (Jiang et al., 2024). Concurrently, when bones are subjected to external forces, fluid flow within the lacuno-canalicular system changes, and the changes that are sensed by osteocytes and converted into biochemical signals, such as the release of signaling molecules like prostaglandin E2 (PGE2) and nitric oxide (NO). These molecules regulate the activity of osteoblasts and osteoclasts, thereby influencing the bone remodeling process (Yang et al., 2024). Through gradient boundary condition modeling, this study reveals the differential effects of loading magnitude (strain), outer wall constraints, and pulsatile blood pressure on intra-osteonal fluid flow behavior, offering a new perspective for understanding mechanical signal transduction mechanisms in the bone microenvironment.

The constraints on the outer wall of the osteon directly influence the driving force of fluid flow by altering the degree of radial deformation. The research findings indicate that as the displacement constraint on the outer wall gradually relaxes from full constraint (radial displacement ≈ 0 μm) to a fully elastic state, the pore pressure, flow velocity, and fluid shear stress all exhibit a significant downward trend (Figures 5–7). This phenomenon is closely associated with the mechanical coupling characteristics between the osteon and the surrounding tissues. When the outer wall is restricted, the pore-pressure gradient generated by the elastic deformation of the bone matrix serves as the main driving force for fluid flow. However, when the constraint is relaxed, the increased radial degree of freedom of the outer wall causes the deformation energy of the matrix to be converted into structural displacement, thereby weakening the pressure gradient for fluid flow. It is worth noting that traditional studies often use simplified boundaries of full constraint or complete freedom, overlooking the actual impact of gradient constraints under physiological conditions (Yu et al., 2023; Evrard et al., 2024). In real-world scenarios, the boundary conditions of the osteon outer wall can be affected by various factors, such as muscle strength, exercise level (muscles tend to remain tightened during regular exercise, compressing the outer wall of bone tissue), or the use of external braces (Kajiwara et al., 2024). This study is the first to quantify the influence of intermediate constraint states (such as 0.01–0.04 μm sinusoidal displacement) on flow parameters. A negative correlation was found between the degree of constraint and the peak pore pressure, flow velocity, and fluid shear stress, revealing the delicate regulatory role of mechanical constraints in the osteon microenvironment.

Pulsatile blood pressure significantly influences osteonal pore pressure through pressure transmission at the inner wall of the Haversian canal, yet has minimal effect on flow velocity and shear stress (Figure 8). As blood pressure increases from no load (A = 0) to severe hypertension (A = 2.5), peak pore pressure rises from 2.7e4 Pa to 6.5e4 Pa, exhibiting a clear dose-response relationship. This phenomenon is attributed to pressure waves generated by periodic vascular dilation directly acting on the osteon’s inner wall and propagating through the pore network to the entire bone matrix (Yu et al., 2025). However, flow velocity does not increase significantly with blood pressure, a finding hypothesized to relate to adaptive regulatory mechanisms in bone tissue: hypertension may maintain fluid flow homeostasis through local permeability adjustments (e.g., dynamic changes in canaliculi pore size) to avoid excessive shear stress-induced damage to osteocytes. Epidemiological studies suggest an association between hypertension and osteoporosis (Huang and Ye, 2024). The results of this study indicate that elevated blood pressure can transmit mechanical signals by increasing pore pressure within the osteon, which may provide a potential biomechanical link for understanding this association. However, it must be recognized that the relationship between hypertension and bone metabolism is complex, likely involving multiple systemic factors such as endocrine, metabolic, and pharmaceutical influences. This study focuses solely on its potential mechanotransduction pathway: sustained high blood pressure may activate osteocyte signaling pathways (e.g., the RANKL/RANK/OPG system) via elevated pore pressure, promoting osteoclast differentiation and enhancing bone resorption (Dutka et al., 2022). Conversely, hypotension-induced inadequate perfusion may inhibit osteoblast metabolism, ultimately disrupting the balance of bone remodeling.

Interstitial fluid flow within osteons is considered a key mediator in skeletal mechanotransduction. When bone deforms under load, the resulting fluid flow and the fluid shear stress it generates have been shown in *in vitro* and *ex vivo* studies to regulate osteocyte activity and signaling molecule release (Wei et al., 2022; Li et al., 2021). This study confirms a direct positive correlation between load intensity and fluid shear stress (Figure 4). As a critical mechanical signal, shear stress activates ion channels (e.g., TRPV4) on osteocyte surfaces and integrin-mediated cytoskeletal signaling, triggering calcium signaling cascades and the MAPK pathway to regulate gene expression related to osteogenesis and osteoclastogenesis (Li et al., 2021; Liu et al., 2023). Notably, the reduction in shear stress (from 2.58 Pa to 1.5 Pa) due to weakened outer wall constraints may diminish the efficiency of mechanical signal transduction. The quantitative results of this study provide specific mechanical context and numerical reference for a deeper understanding of the diminished fluid mechanical stimulus in certain bone loss conditions (e.g., disuse-induced bone loss). This supplements the bone loss explanation framework based on ‘bone mechanosensitivity,’ emphasizing the importance of mechanical environment regulation at the osteon scale (Brent et al., 2021; Buettmann et al., 2020).

Previous studies and the reviewer’s concern highlighted that continuum poroelastic models might overlook the local flow details within the LCS (Verbruggen et al., 2014; Bonivtch et al., 2007). To address this, we performed a coupled multiscale analysis (Figure 10). The results demonstrate that the continuum model serves as an effective “envelope” for the micro-scale fluid dynamics. While the poroelastic model treats the bone matrix as a homogeneous porous medium, predicting a smooth decay of fluid shear stress (FSS) (Figure 10h, red dashed line), the explicit LCS simulation reveals that the actual FSS is discontinuous, characterized by high-magnitude peaks within canaliculi and lows within lacunae. Crucially, our comparison (Figure 10h) establishes a clear link between the two scales: the macro-scale FSS can be viewed as a baseline that, when multiplied by a geometric amplification factor (observed here to be 1.5–2.5 within canaliculi), approximates the peak stimulation on cell processes. This validation confirms that the parametric trends observed in our macro-scale model (e.g., the effects of strain magnitude and outer-wall constraints) are physically valid and can be translated to the cellular level. The macro-model correctly predicts the relative changes in the mechanical environment, which is the primary focus of this study, while the computationally expensive explicit LCS modeling provides the specific absolute magnitudes of local stimuli. Additionally, this study did not explore frequency-dependent effects (e.g., the influence of varying load frequencies on flow parameters), yet bones under physiological conditions experience dynamic loads with broadband characteristics (Yu et al., 2023). Further analysis of the frequency-response relationship will provide critical parameters for optimizing exercise intervention strategies. It should be noted that the prescribed outer-wall displacements are a parametric representation of constraint strength rather than a direct physiological prescription. Future models could incorporate coupled elastic-viscoelastic boundaries to better capture phase relationships between axial loading and radial confinement. Nonetheless, based on the positive correlation between ‘degree of outer wall constraint’ and ‘internal fluid stimulus’ revealed in this study, it inspires the notion that intervention strategies aimed at enhancing or maintaining the mechanical support of tissues surrounding bone (e.g., through specific exercises to improve muscle envelopment and loading of bone, or using devices that provide moderate circumferential support during rehabilitation) could theoretically help maintain a more bone-favorable intraosseous fluid mechanical environment. This provides a mechanical principle-based reference for developing targeted mechanical intervention strategies. For example, periodic mechanical loading devices could increase outer wall constraint strength in long-term bedridden patients to maintain sufficient fluid shear stress. In hypertensive patients, while managing blood pressure, attention should be paid to the potential impact of elevated bone tissue pore pressure on osteocytes, prompting exploration of therapeutic strategies targeting fluid signaling pathways.

The choice to adopt a continuum poroelastic model in this study was based on the following considerations: (1) This study systematically investigates how gradient boundary conditions (e.g., varying outer-wall constraints and pulsatile blood pressure) regulate the global porofluid dynamics of the osteon, rather than the detailed micro-flow within the LCS or mechanotransduction at the individual osteocyte level. The continuum model, while computationally efficient, is sufficient to capture the distribution patterns and trends of macroscopic pore pressure, flow velocity, and fluid shear stress. (2) Explicit modeling of the LCS would drastically increase model complexity and computational burden, especially for the systematic parametric sweep performed here (involving combinations of six outer-wall constraint levels, six blood pressure levels, and five loading magnitudes). The simplified model enabled the completion of numerous simulations within a reasonable timeframe, allowing for a systematic exploration of the independent and coupled effects of each mechanical factor. (3) Our prior model (Yu et al., 2023) focused on the effect of osteocyte orientation on flow and transport within the LCS, necessitating explicit geometric representation. The current model, in contrast, treats the osteon as an integrated functional unit to understand its fluid-mechanical response within a complex mechanical environment. The results provide macro-scale boundary conditions and mechanical inputs for future, more detailed LCS-scale investigations. We acknowledge the crucial role of the LCS in osteocyte mechanosensing and have stated the limitations of our current model in the Discussion. Future work will build upon this continuum framework by incorporating multi-scale coupling methods to integrate the LCS network, thereby providing a more comprehensive understanding of mechanical signal transmission from the osteon to the osteocyte level.

The parametric trends predicted by our model, specifically, the modulation of intra-osteonal fluid flow by gradient loading, pulsatile blood pressure, and radial constraints are corroborated by established *in vitro* and *in vivo* experimental observations, as synthesized below. (1) The simulation result showing a positive correlation between axial strain magnitude (250–5,000 με) and intra-osteonal pore pressure/fluid velocity finds direct support in bone mechanobiology studies. Experiments using rat hindlimb immobilization (a model of skeletal disuse) demonstrate that mechanical unloading leads to reduced cancellous bone mineral density (BMD), disrupted trabecular connectivity, and diminished mechanical stimulation of bone tissue (Kajiwara et al., 2024), which is consistent with the low pore pressure and fluid velocity predicted in our low-strain condition (250 με). Conversely, studies employing belt electrode skeletal muscle electrical stimulation (B-SES) confirm that mechanical stress via muscle contraction can reverse disuse-induced bone microstructural deterioration and enhance bone perfusion-related mechanical signaling (Kajiwara et al., 2024), validating the link between loading intensity and intra-osteonal fluid dynamics. Furthermore, fluid-structure interaction (FSI) modeling of trabecular bone indicates that wall shear stress (WSS) increases with loading magnitude under physiological gait conditions (Wang et al., 2025), providing indirect mechanistic support for our observed strain-dependent fluid flow modulation. (2) Our finding that elevated pulsatile blood pressure predominantly increases pore pressure rather than fluid velocity aligns with the physiology of cardiovascular-osseous crosstalk. The bone vascular system is highly sensitive to systemic blood pressure fluctuations, which directly regulate intraosseous pressure and perfusion distribution by modulating the vasomotor response of bone nutrient arteries (Prisby, 2017). Prior computational work modeling intramedullary pressure effects on Haversian canal fluid flow has yielded results consistent with experimental measurements of intraosseous pressure in animal models (Zhang et al., 2021), reinforcing the physiological relevance of pulsatile blood pressure as a boundary condition. (3) The simulated effect of radial displacement constraints where enhanced constraint increases fluid shear stress is supported by structural and functional studies of the osteonal microenvironment. High-resolution micro-CT imaging of cortical bone reveals that the osteonal outer wall is mechanically integrated with the surrounding lamellar matrix, and the degree of this integration directly influences local strain transmission and fluid flow pathways (van Oers et al., 2008). Biomechanical analyses of human bone samples have identified an inverse relationship between habitual mechanical load (approximated by functional strain) and osteon size (Smith et al., 2022), suggesting that mechanical constraints shape the osteonal microstructure to optimize fluid flow regulation. Additionally, finite element studies of interstitial fluid flow in osteons with spatial gradients of mechanical properties confirm that radial constraints alter the strain field perceived by osteocytes, thereby modulating mechanosensitive signaling cascades (Chen et al., 2024). Collectively, this body of experimental and computational evidence strengthens the physiological plausibility of our model. It underscores that loading magnitude, vascular pressure, and structural constraints are interdependent regulators of the intra-osteonal fluid milieu, ultimately converging on osteocyte mechanotransduction to influence bone adaptation.

In summary, this study systematically analyzed the regulatory mechanisms of the osteon mechanical environment on fluid flow through gradient boundary condition modeling, providing a quantitative framework for investigating mechanical signal transduction in the bone microenvironment and laying a theoretical foundation for clinical mechanical intervention strategies in bone diseases.

## Data Availability

The original contributions presented in the study are included in the article/supplementary material, further inquiries can be directed to the corresponding authors.
